# Choline Supplementation Normalizes Fetal Adiposity and Reduces Lipogenic Gene Expression in a Mouse Model of Maternal Obesity

**DOI:** 10.3390/nu9080899

**Published:** 2017-08-18

**Authors:** Chauntelle Jack-Roberts, Yaelle Joselit, Khatia Nanobashvili, Rachel Bretter, Olga V. Malysheva, Marie A. Caudill, Anjana Saxena, Kathleen Axen, Ahmed Gomaa, Xinyin Jiang

**Affiliations:** 1 Department of Health and Nutrition Sciences, Brooklyn College of the City University of New York, Brooklyn, NY 11210, USA; chauntelle.r@gmail.com (C.J.-R.); yaelle89@yahoo.com (Y.J.); xatia1988@yahoo.com (K.N.); bretterrachel@gmail.com (R.B.); kaxen@brooklyn.cuny.edu (K.A.); ahmedblueboy@hotmail.com (A.G.); 2 Division of Nutritional Sciences, Cornell University, Ithaca, NY 14853, USA; ovm4@cornell.edu (O.V.M.); mac379@cornell.edu (M.A.C.); 3 Department of Biology, Brooklyn College of the City University of New York, Brooklyn, NY 11210, USA; ASaxena@brooklyn.cuny.edu

**Keywords:** choline, fetal adiposity, gestation, obesity, lipogenesis

## Abstract

Maternal obesity increases fetal adiposity which may adversely affect metabolic health of the offspring. Choline regulates lipid metabolism and thus may influence adiposity. This study investigates the effect of maternal choline supplementation on fetal adiposity in a mouse model of maternal obesity. C57BL/6J mice were fed either a high-fat (HF) diet or a control (NF) diet and received either 25 mM choline supplemented (CS) or control untreated (CO) drinking water for 6 weeks before timed-mating and throughout gestation. At embryonic day 17.5, HF feeding led to higher (*p* < 0.05) percent total body fat in fetuses from the HFCO group, while the choline supplemented HFCS group did not show significant difference versus the NFCO group. Similarly, HF feeding led to higher (*p* < 0.05) hepatic triglyceride accumulation in the HFCO but not the HFCS fetuses. mRNA levels of lipogenic genes such as *Acc1*, *Fads1*, and *Elovl5*, as well as the transcription factor *Srebp1c* that favors lipogenesis were downregulated (*p* < 0.05) by maternal choline supplementation in the HFCS group, which may serve as a mechanism to reduce fat accumulation in the fetal liver during maternal HF feeding. In summary, maternal choline supplementation improves indices of fetal adiposity in obese dams at late gestation.

## 1. Introduction

Maternal obesity is a major public health problem which increases the risk of pregnancy complications [1], such as gestational diabetes mellitus (GDM) characterized by hyperglycemia and glucose intolerance, and adverse fetal growth outcomes, such as macrosomia, defined as a birthweight of more than 4 kg [2,3]. Macrosomia at birth has been demonstrated to correlate with higher incidences of adolescent obesity and cardio-metabolic disease later in life [4,5,6].

Moreover, macrosomia resulting from maternal obesity often displays a sheer increase in fat mass without a proportional change in lean tissue mass [7,8]. The rise in fetal adiposity is attributed to both increased de novo lipogenesis and maternal transport of fatty acids to the fetus [9]. Maintaining the homeostasis of lipid metabolism and transport of the maternal-fetal dyad may be an effective approach to prevent macrosomia arising from maternal obesity [10].

Choline is an essential nutrient which participates in various biochemical pathways in the body. Derivatives of choline, e.g., acetylcholine, betaine, and phosphatidylcholine (PC), serve as a neurotransmitter, a methyl donor, and an essential component of cellular and lipoprotein membranes, respectively [11]. Choline deficiency in both humans and rodents results in fatty liver and liver cell damage [12,13]. Choline possibly influences hepatic and whole body lipid metabolism in a multi-faceted manner. Since PC is a component of lipoproteins, its deficiency impairs the secretion of lipoproteins, such as very low density lipoproteins (VLDLs) from the liver, and results in lipid accumulation in the liver [14]. A specific form of PC, 1-palmitoyl-2-oleoyl-sn-glycerol-3-phosphocholine, is a ligand for the peroxisome proliferator-activated receptor alpha (PPARα), which promotes fatty acid catabolism via β-oxidation [15]. Another PC species, dilauroyl-PC, is an agonist of liver receptor homolog 1 (LRH-1), which activates bile acid synthesis [16]. Additionally, intracellular PC synthesis is critical for inhibiting the maturation and activation of sterol regulatory element-binding protein 1 (SREBP1), a transcription factor that promotes lipogenesis [17]. SREBP1 isoform c (SREBP-1c) activates many lipogenic genes including acetyl-CoA carboxylases (ACCs) which convert acetyl-CoA to malonyl-CoA [18], fatty acid synthase (FASN) which uses acetyl-CoA and malonyl-CoA to synthesize fatty acids [19], stearoyl-CoA desaturase-1 (SCD1) and fatty acid desaturases (FADSs) which mediate the formation of unsaturated fatty acids [20], and fatty acid elongase 5 (ELOVL5) which elongates polyunsaturated fatty acids [21]. In summary, choline plays a critical role in enhancing lipid export from the liver, promoting fatty acid catabolism, and reducing lipogenesis. Therefore, maternal choline status may affect circulating lipids supplied to the placenta and eventually acquired by the fetus, whereas choline status in the fetus may influence fetal lipid metabolism directly via the aforementioned mechanisms.

Choline can be oxidized to betaine and serve as a methyl donor for methylation reactions including DNA and histone methylation, thereby modifying the epigenetic marks. Supplementation of different methyl donors (e.g., folate, betaine, choline) consistently alleviates steatosis, suggesting the importance of methylation for lipid metabolism [22,23,24]. An obesogenic diet was shown to alter the DNA methylation profile of FASN in rats [22]. Several other genes related to lipid metabolism, such as PPAR-γ and the LDL receptor, were also reported to be epigenetically regulated via DNA and/or histone methylation [25,26]. Moreover, maternal HF feeding elicited epigenetic alterations in an array of genes related to steatohepatitis in rats [27], while another study demonstrated that maternal folic acid supplementation determined lipid metabolism by altering PPAR expression in rat progeny [28]. As a methyl donor, choline may modify the epigenetic influence of maternal HF feeding on fetal lipid metabolism. 

In this study, we examined the effect of maternal choline supplementation on fetal growth and adiposity at late gestation (i.e., embryonic day E17.5) in HF feeding-induced obese dams. 

## 2. Materials and Methods 

### 2.1. Animals and Diets

**Figure 1:**
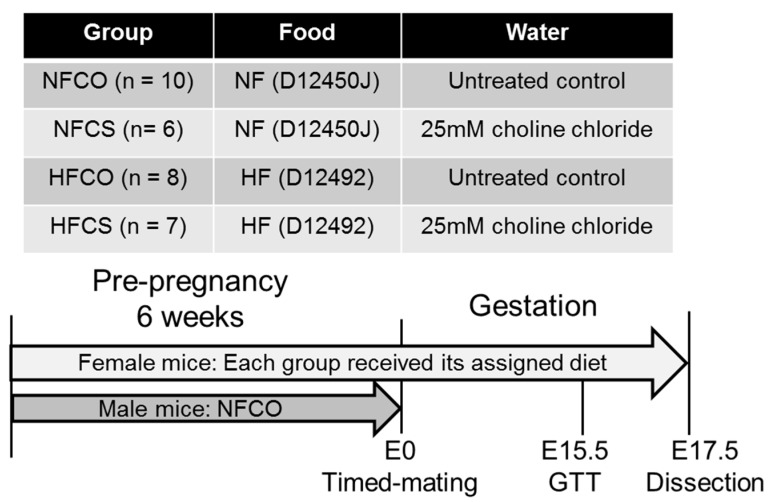
Design of the study. Female C57BL/6J mice were divided into four groups and fed the normal fat (NF) normal choline (CO) diet, NF choline supplemented (CS) diet, HFCO diet, or HFCS diet for 6 weeks before timed-mating and throughout gestation. Male mice followed the NFCO diet until timed-mating. The intraperitoneal glucose tolerance test (GTT) was conducted at embryonic day (E) 15.5 and dissection was conducted at E17.5. *n* is the number of dams in each group from which data and samples were collected.

Six-week-old C57BL/6J mice were purchased from Jackson Laboratories. The mice were kept at a temperature of 22 °C, humidity 40%–60%, and a 12-hour/12-hour light/dark cycle. Regular bedding and enrichment were provided in the cages. Female mice were divided into groups receiving either a high fat diet (HF) or a normal fat control diet (NF). The HF diet contained 60% kcal fat (Research Diets D12492, Research Diets, New Brunswick, NJ, USA) and the NF diet contained 10% kcal fat (Research Diets D12450J). The animals either received 25 mM choline chloride in water (CS) or control untreated drinking water (CO) throughout the study period. Based on the different diets and choline supplementation, the four experimental groups were: NFCO (normal fat, untreated water), NFCS (normal fat, choline supplemented water), HFCO (high fat, untreated water), and HFCS (high fat, choline supplemented water) (Figure 1). Male mice for mating followed the NFCO diet and fluid assignment. All animals had free access to food and water. Composition of experimental diets was previously described by others [29,30]. The HF diet had a higher calorie density than the NF diet (5.24 versus 3.85 kcal/g). Choline bitartrate was added to both diets at a 2 g/4057 kcal basis. Other components in the diets (e.g., lard) also contain choline moieties. We measured the choline content in each non-chemically defined ingredient of the diets and found that the total choline content in the HF diet (10.33 mmol/kg) was estimated to be higher than the NF diet (7.58 mmol/kg) [31].

The mice in each group were fed the assigned diets for six weeks before timed-mating. During timed-mating, each male mouse was placed with two female mice from the same group. The female mouse was removed from mating when a vaginal plug was present, and the day was recorded as E0.5. Female mice continued on their assigned diets during gestation until E17.5. Food and water intake, as well as the weight of each animal was measured weekly. Choline intake was calculated as the sum of choline consumed via food and water, based on the concentration of choline in food and water multiplied by the weight and volume consumed. Feeding was conducted on an equal number of female mice in each group (*n* = 10) but not every animal conceived successfully. The final number of dams in each group was: 10 (NFCO), 6 (NFCS), 8 (HFCO), and 7 (HFCS).

### 2.2. Intraperitoneal Glucose Tolerance (IGT) Tests

At E15.5, the animals were fasted for 6 hours starting from the beginning of the light cycle. To conduct the IGT tests, 20% d-glucose (2 g glucose/kg body weight) was injected into the peritoneum of the mice. A drop of blood was obtained via tail nicking to measure blood glucose using a glucometer at baseline (0 min), 15, 30, 60, 90, and 120 min after glucose injection. The Area Under the Curve (AUC) was calculated [32].

### 2.3. Tissue Collection

At E17.5, the mice were euthanized after six hours of food deprivation. Cardiac puncture was performed instantly after euthanasia of the female mice to collect whole blood samples in a serum separator tube (BD, Franklin Lakes, NJ, USA). The blood was centrifuged at 12,000× *g* for 10 min to obtain serum. Maternal visceral fat and livers were dissected and weighed. Fetuses were retrieved, rinsed with phosphate-buffered saline (PBS), dried with absorbent paper, and weighed with an analytical scale. Thereafter, the fetal carcasses were dissected to retrieve whole liver and brain samples. The livers, brains, and fetal carcasses were immersed in RNAlater^®^ (Thermo Scientific, Grand Island, NY, USA) for overnight stabilization or snap frozen in liquid nitrogen before being stored at −80 °C. The study protocol was approved by the Institutional Animal Care and Use Committee (IACUC) at Brooklyn College. The animal study approval number is 274.

### 2.4. Analytical Measurements

For analytical measurement of placental samples, we excluded dams with a litter size lower than 5 or higher than 10 to prevent the potential confounding effect of litter size on metabolic parameters. Two samples were randomly selected from each litter unless specified otherwise. Litter size or breeding success rate was not affected by group assignment (data not shown).

#### 2.4.1. Embryo Sexing

Sex of all fetuses was determined by PCR of a sequence specific to the mouse sex-determining region Y (Sry) gene on the Y chromosome [33]. 

#### 2.4.2. Fetal Adiposity Assessment

Total fat of fetuses was extracted using the Folch method [34]. Briefly, carcasses (without liver and brain) were pulverized in liquid nitrogen. A portion of about 50 mg of the carcass was weighed with an analytical balance and immersed in 0.9% sodium chloride. The samples were homogenized with a homogenizer. Thereafter, 800 μL of chloroform: methanol (2:1) was added to the samples and mixed thoroughly on a shaker for one hour. The mixture was centrifuged at 5000× *g* for 10 min after which the lower layer of liquid was transferred to a pre-weighed microcentrifuge tube and dried using a vacuum dryer. After the sample was completely dried in the pre-weighed tube, it was again weighed. The difference between two weight measurements was the weight of fat extracted from the sample. Percent total fat was calculated as fat weight/carcass sample weight × 100%.

#### 2.4.3. Serum Insulin, Glucose, Triglyceride, and Free Fatty Acid (FFA) Measurements

Maternal serum biomarkers were measured with assay kits according to the manufacturers’ instructions: insulin was measured with the Mouse Insulin ELISA kit (ALPCO, Salem, NH, USA); glucose and triglycerides were measured with the glucose and triglyceride colorimetric assay kits (Cayman, Ann Arbor, MI, USA); FFAs were measured with the HR Series NEFA-HR(2) colorimetric reagents (Wako Diagnostics, Richmond, VA, USA).

#### 2.4.4. Homeostasis Model Assessment of Insulin Resistance (HOMA-IR) Index

The HOMA-IR index is an assessment of insulin resistance. It was calculated as [fasting serum glucose (mmol/L) × fasting serum insulin (pmol/L)/22.5].

#### 2.4.5. Choline Measurements

Whole fetal livers were used for choline extraction and quantification. Measurements of choline and its derivatives were conducted using LC-MS/MS methodology [35]. The liver of one fetus from each dam was randomly chosen for the measurement.

#### 2.4.6. RNA Extraction and Quantitative Real-Time PCR

RNA was extracted from fetal livers using the TRIzol® reagent (Thermo Fisher Scientific, Waltham, MA, USA). Reverse transcription was conducted using the High-Capacity cDNA Reverse Transcription kit (Thermo Fisher Scientific, Waltham, MA, USA) following the manufacturer’s instructions. Gene transcript abundance was analyzed by quantitative real-time PCR with SYBR green detection using the CFX384 Touch™ Real-Time PCR Detection System (Bio-Rad, Hercules, CA, USA). The reaction conditions were as follows: 95 °C for 3 min, followed by 39 cycles with 20 s at 95 °C, 30 s at 58 °C, and 20 s at 72 °C. Data were normalized as the fold difference of the gene of interest relative to the housekeeping gene beta-actin (Actb) and analyzed using the ΔΔCt method [36]. Primers were either designed using GeneRunner Version 3.01 (http://www.softpedia.com) (Table S1) or previously published [37]. Expression of the following genes was analyzed: *Acc1*, *Acc2*, peroxisomal acyl-coenzyme A oxidase 1 (*Acox1*), betaine-homocysteine *S*-methyltransferase 1 (*Bhmt1*), choline dehydrogenase (*Chdh*), carbohydrate-responsive element-binding protein (*Chrebp1*), diacylglycerol *O*-acyltransferase 1 (*Dgat1*), *Elovl5*, *Fads1*, *Fasn*, fatty acid transport protein 1 (*Fapt1*), microsomal triglyceride transfer protein (*Mttp*), choline-phosphate cytidylyltransferase A (*Pcyt1a*), phosphatidylethanolamine *N*-methyltransferase (*Pemt*), and *Scd1*.

#### 2.4.7. Maternal and Fetal Liver Triglyceride Measurements

Whole maternal livers were pulverized in liquid nitrogen. Thereafter, 50 mg of the pulverized tissue was used for triglyceride quantification using the Triglyceride Colorimetric Assay Kit (Cayman) according to the manufacturer’s instructions. Whole fetal livers were used for triglyceride concentration quantification using the same method.

#### 2.4.8. Statistical Analyses

General linear models (GLMs) were constructed to assess the differences in the dependent variables (e.g., embryonic weight and gene expression) with diet (D: HF or NF), supplementation (S: CO or CS), and their interaction (D × S) as independent variables. GTT curves were analyzed with GLM Repeated Measure. Posteriori analyses were conducted to detect differences among the groups using one-way analysis of variance (ANOVA) followed by post-hoc Fisher's least significant difference (LSD) tests if there was a D × S interaction. Considering that the small sample size may limit the statistical power to detect a true interaction, *p_d×s_* ≤ 0.2 was used to identify suggestive evidence for the presence or absence of interaction. This method was successfully used in other studies [38,39]. For the dependent variables that included measurements of multiple embryos from the same dam (e.g., embryonic weight, body fat), we first used GLMs to assess the effect of embryonic sex adjusted for D and S. Since no sexual dimorphism was detected, we calculated the averages of each dam and use the pooled data from each dam for GLM and posteriori ANOVA analyses. Litter size was also included in the model as an independent variable if it significantly modified the dependent variable. Dependent variables deviating from the normal distribution were logarithmically transformed before analysis. A *p* value < 0.05 was considered as significant. Values are presented as means ± standard error of mean (SEM).

## 3. Results

### 3.1. Food and Choline Intake

The weight of food pellets consumed each week was lower (*p_d_* < 0.01) in the HF groups than in the NF groups (Table 1). However, since the HF diet had a higher calorie density than the normal diet, the HF groups still had higher (*p_d_* < 0.01) caloric intake per week than the NF groups. There was a D × S interaction for water consumption (*p_d×s_* = 0.02). However, posteriori analysis did not reveal significant difference among the groups (*p* = 0.1). Total choline intake from food and water was on average 4.5 times higher in the choline supplemented groups versus the control groups. Total choline intake from food and water was not significantly different between the HFCS and NFCS groups. The HFCO group had about 21% higher (*p* < 0.01) weekly choline consumption from food than the NFCO group. However, the amount of additional choline from food was relatively small compared to the supplemental dose from water, and was not likely to confound the effect of choline supplementation on the NF and HF dams.

### 3.2. Maternal Weight, Abdominal Adiposity, Glucose Tolerance, and Serum Biomarkers

**Figure 2:**
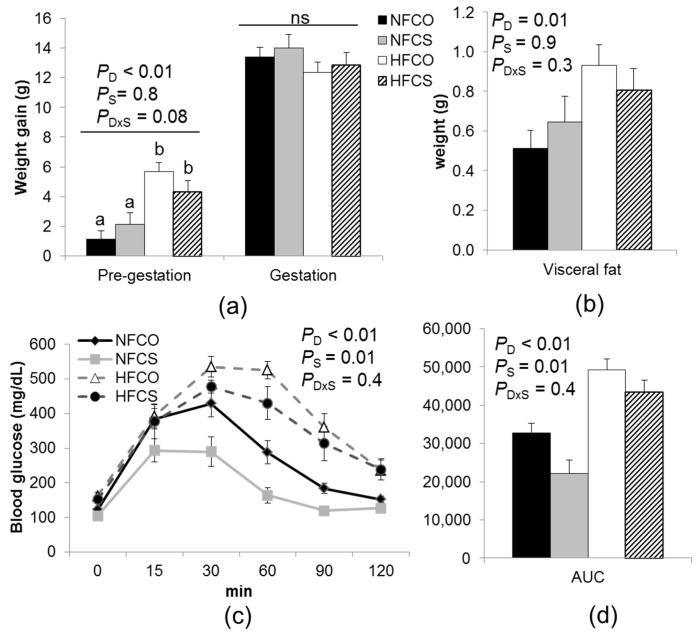
Weight gain and intraperitoneal glucose tolerance (IGT) of dams fed experimental diets. (**a**) Weight gain was measured both before timed-mating and during gestation (E0.5–E17.5); (**b**) Visceral fat was measured at E17.5; (**c**) IGT tests were conducted at E15.5 with 2 g/kg D-glucose injected; (**d**) The area under the curve (AUC) of the IGT tests. NFCO (solid bars): *n* = 10; NFCS (shaded bars): *n* = 6; HFCO (open bars): *n* = 8; HFCS (hatched bars): *n* = 7. Data were analyzed using the general linear model. Posteriori analysis of variance (ANOVA) followed by posthoc Fisher‘s least significant difference (LSD) analysis between groups was conducted with *p_d×s_* ≤ 0.2. Values are mean ± standard error of mean (SEM); different letters indicate *p* < 0.05 in the posthoc analysis. ns: *p_d_*, *p_s_*, and *p_d×s_*, not significant. CO: control; CS: choline supplemented; D: diet; HF: high-fat diet; NF: normal-fat diet; S: supplementation.

As anticipated, the HF groups had significantly higher (*p_d_* < 0.01) average weight gain than the NF groups before gestation (Figure 2a). However, maternal weight gain during gestation (E17.5 versus E0.5) did not differ among the groups. Choline supplementation did not modify weight gain. Similarly, the HF groups had higher (*p_d_* = 0.01) visceral adiposity versus the NF groups, which was not modified by maternal choline supplementation (Figure 2b).

HF feeding led to impaired glucose tolerance of pregnant mice at E15.5 (HF versus NF groups, *p_d_* < 0.01) (Figure 2c,d), suggesting that blood glucose disturbances, typically observed in GDM, were maintained into late gestation. Interestingly, choline supplementation improved glucose tolerance of the dams (*p_s_* < 0.01). Maternal HF feeding tended to interact with choline supplementation to affect insulin secretion (*p_d×s_* = 0.1). Posteriori analysis suggested that maternal serum insulin levels were higher in the NFCS than the other groups (*p* = 0.02) (Table 2), suggestive of hyperinsulinemia that is often associated with insulin resistance. However, neither the fasting serum glucose nor the HOMA-IR index was significantly affected by maternal HF or CS. Collectively, the NFCS dams may have differential regulation of insulin secretion or blood glucose uptake at E17.5, but the mechanism and the combined effect of different factors on their blood glucose control are unclear. Choline supplementation led to higher (*ps* = 0.02) serum triglyceride concentrations and tended to increase (*ps* = 0.06) serum FFA levels versus the control (NFCO and HFCO) groups, which could be related to increased fat export from the liver. Indeed, choline supplementation significantly reduced liver triglyceride levels (*ps* < 0.01). In summary, maternal biomarkers did not seem to be consistently improved by choline supplementation.

### 3.3. Placental and Embryonic Outcomes 

#### 3.3.1. Maternal Choline Supplementation during HF Feeding Did Not Affect Fetal Weight but Reduced Fetal Adiposity 

**Figure 3:**
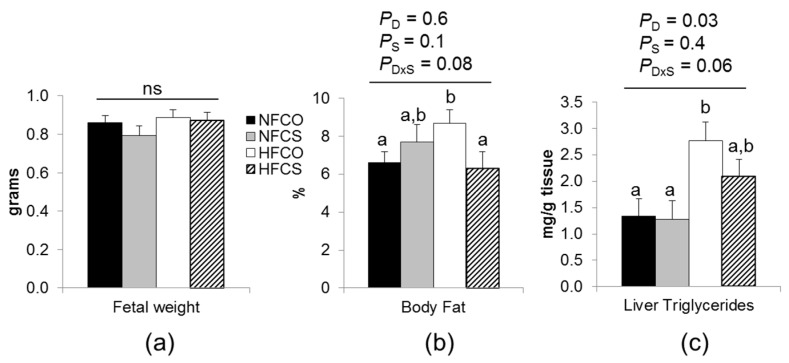
Fetal weight and adiposity at E17.5. (**a**) Fetal weight; (**b**) percent total body fat of fetuses; (**c**) fetal liver triglyceride concentrations. Experimental diets were fed to dams from 6 weeks before timed-mating to gestational day 17.5. Data were analyzed using the general linear model. Posteriori analysis of variance (ANOVA) followed by posthoc Fisher‘s least significant difference (LSD) analysis between groups was conducted with *p_d×s_* ≤ 0.2. NFCO (solid bars): *n* = 9; NFCS (shaded bars): *n* = 6; HFCO (open bars): *n* = 7; HFCS (hatched bars): *n* = 6. *n* is the number of dams. Fetal weight was analyzed with pooled data of all fetuses in each dam (5–10/dam); body fat and liver triglyceride concentrations were analyzed with pooled data of two randomly selected fetuses in each dam. Values are mean ± standard error of mean (SEM); different letters indicate *p* < 0.05 in the posthoc analysis. ns: *p_d_*, *p_S_*, and *p_d×s_*, not significant. CO: control; CS: choline supplemented; D: diet; HF: high-fat diet; NF: normal-fat diet; S: supplementation.

At E17.5, fetal weight was not significantly different among the groups (Figure 3a). Despite the lack of difference in body weight, maternal HF feeding and choline supplementation tended to interact to affect percent body fat (*p_d×s_* = 0.08) and hepatic triglyceride content (*p_d×s_* = 0.06) of the fetuses. Posteriori analysis suggested that percent body fat of the fetus was higher in the HFCO group (HFCO versus NFCO, *p* = 0.03), whereas the HFCS group had similar percent body fat to NFCO (Figure 3b). Moreover, the HFCO group also had higher hepatic triglyceride content (*p* = 0.02), whereas that of the HFCS group was not significantly different from the NFCO group (Figure 3c). These results suggested that maternal choline supplementation partially prevented excess adiposity of fetuses from the HF-fed dams at E17.5.

#### 3.3.2. Maternal Choline Supplementation during HF Feeding Downregulated Lipogenic Gene Expression in Fetal Livers

**Figure 4:**
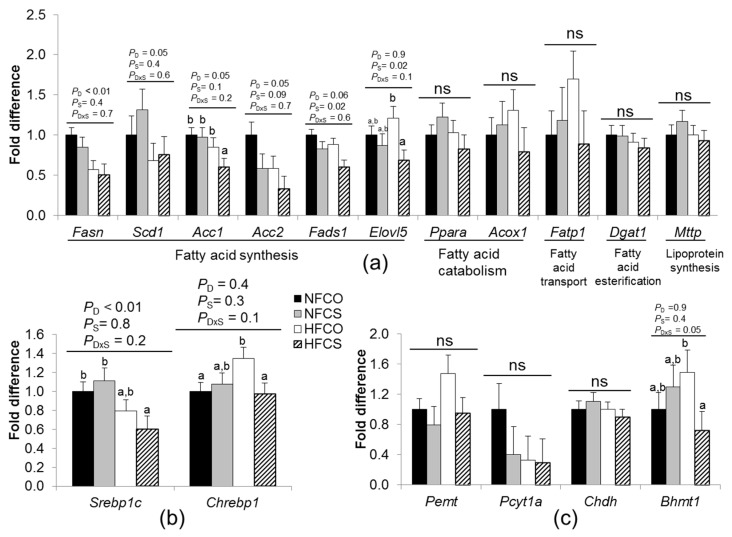
Fetal liver mRNA abundance at E17.5. Experimental diets were fed to dams from 6 weeks before timed-mating to gestational day 17.5. (**a**) Genes involved in lipid metabolism; (**b**) transcription factors; (**c**) genes involved in choline metabolism. mRNA levels were measured by real-time PCR. Data were analyzed using the general linear model. Data were analyzed using the general linear model. Posteriori analysis of variance (ANOVA) followed by posthoc Fisher's least significant difference (LSD) analysis between groups was conducted with *p_d×s_* ≤ 0.2. NFCO (solid bars): *n* = 9; NFCS (shaded bars): *n* = 6; HFCO (open bars): *n* = 7; HFCS (hatched bars): *n* = 6. *n* is the number of dams. Data from two fetuses in each dam were pooled and included in the analysis. Values are mean ± standard error of mean (SEM); different letters indicate *p* < 0.05 in the posthoc analysis; ns: *p_d_*, *p_S_*, and *p_d×s_*, not significant. *Acc*: acetyl-CoA carboxylase; *Acox1*: peroxisomal acyl-coenzyme A oxidase 1; *Bhmt*: betaine-homocysteine *S*-methyltransferase; *Chdh*: choline dehydrogenase; *Chrebp1*: Carbohydrate-responsive element-binding protein; *Dgat1*: diacylglycerol O-acyltransferase 1; *Elovl5*: fatty acid elongase 5; *Fasn*: fatty acid synthase; *Fatp1*: fatty acid transport protein 1; *Mttp*: microsomal triglyceride transfer protein; *Pcyt1a*: choline-phosphate cytidylyltransferase A; *Pemt:* phosphatidylethanolamine N-methyltransferase; *Ppara*: Peroxisome proliferator-activated receptor alpha; *Scd1*: stearoyl-CoA desaturase-1; *Srebp1*: Sterol regulatory element-binding protein 1; CO: control; CS: choline supplemented; D: diet; HF: high-fat diet; NF: normal-fat diet; S: supplementation.

We next examined the mRNA abundance of a series of genes related to lipid metabolism in the fetal liver (Figure 4a). HF feeding decreased mRNA abundance of *Fasn* (HF versus NF, *p_d_* < 0.01), an enzyme that mediates the production of fatty acids from acetyl-CoA, *Acc2* (*p_d_* = 0.05) which also mediates fatty acid synthesis, and *Scd1* (*p_d_* = 0.05), a rate-limiting enzyme in the formation of monounsaturated fatty acids. Maternal choline supplementation decreased *Fads1* which is involved with unsaturated fatty acid synthesis by mediating fatty acid desaturation (CS versus CO, *p_s_* = 0.02). Maternal HF feeding and choline supplementation tended to interact to affect the expression of *Acc1* which participates in fatty acid synthesis and *Elovl5* which affects unsaturated fatty acid elongation. Posteriori analysis suggested that their expression levels were both decreased by maternal choline supplementation during HF feeding (*Acc1*: HFCS versus the other three groups, *p* < 0.05; *Elovl5*: HFCS versus HFCO, *p* = 0.01).

Genes related to other pathways of lipid metabolism, including *Ppara* and *Acox1* which mediate β-oxidation of fatty acid catabolism, *Fatp1* which helps fatty acid transport, *Dgat1* which mediates the esterification reaction of triglyceride synthesis, or *Mttp* which assists with lipoprotein assembly were not differentially expressed due to HF feeding or maternal choline supplementation (*p* > 0.05) (Figure 4a).

SREBP-1c and ChREBP1 are transcription factors that upregulate lipogenesis. Maternal HF feeding and choline supplementation tended to interact to affect their expression (Figure 4b). Posteriori analysis suggested that *Srebp1c* mRNA levels were lower in the HFCS versus NFCO group (*p* = 0.02), while there was no significant difference in its gene expression between the HFCO and NFCO groups (Figure 4b). *Chrebp1* was upregulated only in the HFCO group (*p* = 0.03), but not in the HFCS group when compared to NFCO. Collectively, both the decrease in *Srebp1c* and the lack of increase in *Chrebp1* mRNA abundance due to maternal choline supplementation may facilitate attenuation of hepatic lipogenesis during HF feeding.

#### 3.3.3. HF Feeding and Maternal Choline Supplementation Altered Fetal Hepatic Choline Metabolism

We then examined choline metabolite concentrations in the fetal liver (Table 3). HF feeding led to 17% lower free choline content versus NF (*p_d_* = 0.05). Conversely, maternal choline supplementation tended to increase (*p_s_* = 0.07) free choline concentrations. Maternal choline supplementation increased fetal liver betaine content by 80% (CS versus CO, *p* < 0.01), especially in the HFCS group which had higher betaine content than the other three groups (*p* < 0.05). Other fetal liver choline metabolites were not altered by HF feeding or choline supplementation. 

Lastly, we examined whether the expression of genes involved in choline metabolism was altered by the different diets. Maternal HF feeding and choline supplementation interacted (*p_d×s_* = 0.05) to affect the mRNA levels of *Bhmt1* which mediates methyl group donation from betaine to homocysteine. *Bhmt1* expression was decreased by maternal choline supplementation during HF feeding (70% decrease, HFCS versus HFCO, *p* = 0.03). Previous studies suggest that BHMT1 is downregulated by *S*-adenosylmethionine which is derived from methionine, a reaction product of BHMT1 [40]. Both the decrease in *Bhmt1* expression and increase in betaine content in the HFCS group suggest an enhanced methylation activity. Other genes involved in choline metabolism, including *Pemt*, *Chdh*, and *Pcyt1a*, were not altered by HF feeding or choline supplementation. 

## 4. Discussion

In the current study, we investigated the influence of maternal choline supplementation on fetal adiposity and hepatic lipid metabolism at E17.5 (late gestation) in mice fed a HF diet to induce maternal obesity. We have found that HF feeding increased fetal adiposity and hepatic fat accumulation, whereas maternal choline supplementation attenuated these abnormalities and downregulated the expression of lipogenic genes in the fetal liver.

It is well established that choline deficiency results in steatosis, whereas choline and betaine supplementation improves non-alcoholic fatty liver disease (NAFLD) in adult animals [24]. We report here for the first time that maternal choline supplementation before and during gestation prevented excess adiposity and hepatic triglyceride over-accumulation in fetuses from HF-fed mouse dams. These results are consistent with the proposed model where choline influences macronutrient metabolism and facilitates the maintenance of metabolic homeostasis [41], especially under the condition of an environmental challenge (e.g., HF feeding). 

In the current model of maternal HF feeding, we observed higher pre-pregnancy weight gain and impaired glucose tolerance of dams at gestation day 15.5, which resembles GDM resulting from maternal obesity in humans. Consistent with a previous report, fetal weight was not significantly different between the HF and NF groups at E17.5 [10]. However, similar to the previous study [10], the HFCO fetuses not only had higher percent total body fat, but also had higher triglyceride concentrations in the liver versus NFCO. The mouse fetus acquires fat predominantly from maternal transport but can also conduct de novo lipogenesis [9,42]. Although fat acquisition accelerates at late gestation, the mouse fetus contains less than 10% of body weight as fat at birth. Nevertheless, adiposity at birth is a good indicator of adiposity and glucose intolerance in adulthood [1]. As such, preventing excess body fat accumulation at term may have positive implications for metabolic balance throughout the life-course. In this study, maternal choline supplementation seems to be an effective way to prevent excess adiposity, since the HFCS fetus had comparable whole body adiposity and hepatic fat content to NFCO.

The potential mechanism through which maternal choline supplementation helps normalize fetal fat metabolism may be multi-faceted. Maternal choline supplementation improved fetal choline status as was demonstrated by the higher betaine concentrations and the trend of higher free choline concentrations in fetal livers in the present study. Increasing choline availability helps maintain integrity of cellular membranes, provide structural materials for lipoproteins, provide ligands (PPAR-α, LRH-1) or suppressors of transcription factors (SREBP-1c) that influence lipid metabolism [15,16,17], and supply methyl groups for epigenetic control of lipid metabolic genes of the fetus [22,41,43]. Maternal choline supplementation may also affect maternal lipid metabolism, thereby altering placental uptake and transport of lipids to the fetus. However, the current study suggested that maternal choline supplementation increased lipid concentrations in maternal circulation, which was not consistent with the lower lipid accretion of the HFCS versus the HFCO fetuses. Examination of the placenta will provide more insights into the regulation of lipid transport to the fetus in response to HF feeding and choline supplementation and help discern the discrepancy.

We explored the expression of genes related to fatty acid synthesis, esterification, degradation, transport, and assembling into lipoprotein in the fetal liver. Downregulation of certain lipogenic genes including *Fasn*, *Scd1*, and *Acc2* in the fetal liver seemed to be a common response to maternal HF feeding regardless of choline exposure. However, these observations directly contrasted with what was found in studies of adult mice, in which HF feeding increased both *Fasn* and *Scd1* expression [44,45]. These inconsistencies may be related to the adjustment in maternal transport of lipids or differential regulation of the acquired fat by the fetal liver, which requires further investigation. We further observed that maternal choline supplementation in HF dams decreased expression of other fatty acid synthesizing genes, including *Acc1*, *Elovl5*, and *Fads1*. The additional decrease in de novo lipogenic gene expression in the HFCS versus HFCO fetuses may further diminish fatty acid synthesis, thereby compensating for the increased influx of lipids from the HF dams.

The main mechanism by which choline exerts its impact on lipogenesis remains to be elucidated. One potential mediator is SREBP-1c, a transcription factor that upregulates a series of lipogenic genes including those we examined. The HFCS group had lower *Srebp1c* expression compared with the NFCO group whereas the HFCO group did not downregulate the expression of this gene. Maintenance of cellular membrane PC is necessary to prevent SREBP-1c from translocating to the nucleus and functioning as a transcription factor [17]. Although PC content in the fetal liver was not significantly different among the groups, maternal HF feeding decreased free choline content of fetal livers, which could be a result of increased use of choline, possibly to synthesize PC. Maternal choline supplementation tended to increase fetal hepatic free choline status thereby counteracting the effect of HF feeding. 

ChREBP1, a transcription factor that gets activated by hyperglycemia, also upregulates lipogenesis [46]. *Chrebp1* expression increased in the HFCO fetal liver but not in the HFCS fetal liver when compared to NFCO animals. This may also contribute to the observed lower lipogenic gene expression in the HFCS group. However, since we do not have data about fetal glucose levels, we cannot conclude that better fetal glycemic control in the HFCS group prevented the elevation in *Chrebp1* expression. 

The decrease in de novo lipogenic gene expression does not preclude other possible mechanisms by which choline regulates fetal fat metabolism. Therefore, we examined the expression of fetal hepatic *Mttp* which participates in lipoprotein assembly, as well as *Ppara* and *Acox1* which participate in lipid catabolism, yet did not find significant differences among the four groups. A previous study [47] suggested that choline deficiency exacerbated fatty liver in HF fed mice but improved glucose tolerance. The authors speculated that choline deficiency decreased the use of diacylglycerol for phosphatidylcholine synthesis, thereby sparing hepatic diacylglycerol for FFA esterification. The increased use of FFAs for esterification reduced the levels of circulating FFAs, a known risk factor for impaired glucose tolerance [47]. However, we did not find any changes in expression of *Dgat1*, which mediates the last step of esterification to form triglycerides, among the groups either. Since we did not retrieve blood from the fetuses, we could not quantify and determine whether FFAs or lipoproteins were lower in the circulation of HFCS fetuses compared with HFCO. It should be noted that the difference in fetal fat accumulation could also be a result of differential maternal transport of lipids. Although the triglyceride concentrations in maternal circulation were higher in the HFCS versus HFCO group, the HFCS fetuses had lower adiposity. Whether it was due to reduced placental lipid uptake or transport remains to be discerned.

Another significant difference between the HFCS and HFCO fetal livers relates to their betaine content, suggesting that the epigenetic regulation of hepatic genes could be affected by the different availability of this methyl donor for DNA and histone methylation. The lower expression of *Bhmt1* in the HFCS group versus the NFCO group may also indicate an ample supply of betaine as the substrate for methyl group donation and generation of *S*-adenosylmethionine, because *S*-adenosylmethionine provides feedback inhibition which downregulates BHMT [40]. Supplementation of betaine to the maternal diet has been shown to enhance DNA methylation of genes involved in cholesterol metabolism in neonatal piglets [26]. The genes that we observed to be downregulated in the HFCS group, such as *Srebp1c* and *Fads1*, have also been reported to be regulated by DNA methylation in other studies [48,49]. Further study is needed to quantify DNA methylation of the lipogenic genes to confirm the role of epigenetics in controlling their expression in the current model.

Our study provides supportive evidence that maternal choline supplementation normalizes fetal adiposity and reduces lipogenic gene expression in HF fed mice at term. Although we do not have corresponding protein levels for the genes, based on previous studies [50,51], mRNA abundance assessed by real-time PCR seems to be a sensitive method to detect subtle transcriptional changes that are induced by dietary factors. The consistent decreases in the expression of a group of lipogenic genes, in combination with the phenotypic difference in fat content, further strengthen our mechanistic model. Future studies should be directed in further delineating mechanisms by which maternal choline supplementation modulates fetal response to maternal HF feeding.

## 5. Conclusions

In conclusion, during HF feeding, maternal choline supplementation prevents excess adiposity and hepatic triglyceride overload in mouse fetuses. Downregulation of lipogenic gene expression in the fetal liver is a possible mechanism by which fetal adiposity is attenuated in the HFCS animals.

## Figures and Tables

**Table 1:** Average weekly food and choline intake of dams ^1^.

Food and Choline Intake	NFCO	NFCS	HFCO	HFCS	*p* Value		
	(*n* = 10)	(*n* = 6)	(*n* = 8)	(*n* = 7)	D	S	D × S
Food intake (g/week)	17.4 ± 0.2	17.4 ± 0.3	15.5 + 0.3	14.9 ± 0.3	<0.01	0.3	0.4
Caloric intake (kcal/week)	67 ± 1	66 ± 2	81 ± 1	78 ± 1	<0.01	0.3	0.3
Water intake (mL/week)	20.2 ± 0.4 ^a^	21.7 ± 0.8 ^a^	21.2 ± 0.4 ^a^	20.2 ± 0.5 ^a^	0.6	0.7	0.02
Choline intake from food (µmol/week)	132 ± 2	133 ± 4	160 ± 3	155 ± 3	<0.01	0.5	0.4
Choline intake from water (µmol/week)	N/A	542 ± 16	N/A	501 ± 13	0.08	N/A	N/A
Total choline intake (µmol/week)	132 ± 2 ^a^	663 ± 12 ^c^	160 ± 3 ^b^	664 ± 12 ^c^	0.4	0.01	0.1

^1^ Experimental diets were fed to dams from 6 weeks before timed-mating to gestational day 17.5. *n* is the number of dams. Data were analyzed using the general linear model. Posteriori analysis of variance (ANOVA) followed by posthoc Fisher's least significant difference (LSD) analysis between groups was conducted with *p_d×s_* ≤ 0.2. Different letters: a, b, and c, indicate *p* < 0.05 in the posthoc analysis. Values represent means ± standard error of mean (SEM). CO: control; CS: choline supplemented; D: diet; HF: high-fat diet; NF: normal-fat diet; N/A, not applicable; S: supplementation.

**Table 2:** Maternal serum and liver biomarkers at gestational day 17.5 ^1^.

Maternal Biomarkers	NFCO	NFCS	HFCO	HFCS	*p* Value		
	(*n* = 8)	(*n* = 6)	(*n* = 8)	(*n* = 7)	D	S	D × S
Serum insulin (ng/mL)	0.63 ± 0.05 ^a^	0.81± 0.06 ^b^	0.57 ± 0.05 ^a^	0.57 ± 0.05 ^a^	0.01	0.08	0.1
Serum glucose (mg/dL)	103.5 ± 5.9	88.9± 6.9	103.7 ± 5.9	94.1 ± 6.4	0.7	0.07	0.7
HOMA-IR index	28.0 ± 2.9	30.3 ± 3.4	24.9 ± 2.9	23.2 ± 3.1	0.1	0.9	0.5
Serum triglycerides (mg/dL)	37.9 ± 3.5	46.6 ± 5.2	31.2 ± 3.7	50.6 ± 4.1	0.7	<0.01	0.5
Serum FFAs (mmol/L)	0.52± 0.12	0.74 ± 0.2	0.64 ± 0.14	0.80 ± 0.16	0.9	0.06	0.4
Liver triglycerides (mg/g tissue)	17.8 ± 4.9 ^a^	9.3 ± 6.4 ^a^	37.3 ± 5.5 ^b^	13.9 ± 5.5 ^a^	0.04	<0.01	0.2

^1^ Experimental diets were fed to dams from 6 weeks before timed-mating to gestational day 17.5. *n* is the number of dams. Data were analyzed using the general linear model. Posteriori analysis of variance (ANOVA) followed by posthoc Fisher’s least significant difference (LSD) analysis between groups was conducted with *p_d×s_* ≤ 0.2. Different letters: a and b, indicate *p* < 0.05 in the posthoc analysis; Values represent means ± standard error of mean (SEM). FFA: free fatty acid; HOMA-IR: homeostasis model assessment of insulin resistance = fasting insulin concentration (pmol/L) × fasting glucose concentration (mmol/L)/22.5. CO: control; CS: choline supplemented; D: diet; HF: high-fat diet; NF: normal diet; S: supplementation.

**Table 3:** Choline-derived metabolite concentrations in the fetal liver at gestational day 17.5 ^1^.

Metabolites (nmol/g Tissue)	NFCO	NFCS	HFCO	HFCS	*p* Value		
	(*n* = 9)	(*n* = 6)	(*n* = 7)	(*n* = 6)	D	S	D × S
Choline	172 ± 12	196 ± 13	144 ± 13	169 ± 13	0.05	0.07	0.9
Betaine	719 ± 89 ^a^	1290 ± 102 ^b^	957 ± 103 ^a^	1740 ± 103 ^c^	<0.01	<0.01	0.2
Methionine	139 ± 12	158 ± 14	134 ± 14	134 ± 14	0.3	0.5	0.5
Lysophosphatidylcholine	253 ± 28	235 ± 33	273 ± 32	177 ± 33	0.6	0.08	0.3
Glycerophosphocholine	633 ± 60	735 ± 70	610 ± 70	587 ± 69	0.2	0.6	0.4
Phosphocholine	537 ± 54	687 ± 62	486 ± 62	629 ± 62	0.02	0.4	0.9
Phosphatidylcholine	22,006 ± 914	21,511 ± 1055	21,515 ± 1055	19,317 ± 1055	0.2	0.2	0.4
Sphingomyelin	3707 ± 228	3423 ± 263	3794± 263	3474 ± 263	0.8	0.2	0.9

^1^ Experimental diets were fed to dams from 6 weeks before timed-mating to gestational day 17.5. *n* is the number of fetuses. Livers of 6–9 fetuses from different dams (1 fetal liver/dam) were randomly chosen from each group for quantification. Data were analyzed using the general linear model. Posteriori analysis of variance (ANOVA) followed by posthoc Fisher’s least significant difference (LSD) analysis between groups was conducted with *p_D×S_* ≤ 0.2. Different letters: a, b, and c, indicate *p* < 0.05 in the posthoc analysis; Values represent means ± standard error of mean (SEM). CO: control; CS: choline supplemented; D: diet; HF: high-fat diet; NF: normal-fat diet; S: supplementation.

## Supplementary Materials

The following are available online at www.mdpi.com/2072-6643/9/8/899/s1, Table S1: Primers used for real-time PCR.
