# Supplementary material for: Choline Supplementation Normalizes Fetal Adiposity and Reduces Lipogenic Gene Expression in a Mouse Model of Maternal Obesity
Source: Nutrients. 2017 Aug 18;9(8):899. doi: 10.3390/nu9080899 (PMC5579692; doi:10.3390/nu9080899)
Supplement: Supplementary file 1 [file nutrients-09-00899-s001.pdf]

Supplementary Information

Table S1. Primers used for real-time PCR[1]

	Forward primer	Reverse primer
Acc1	5' GGAGCAGAGAACCTTCGGGG 3'	5' CGGACAAGGTAAGCCCCAAT 3'
Acc2	5' AGGCAGTCCGCAGGGTGATG 3'	5' CCGCCTTACGCTGCAGGGTG 3'
Actb	5' TAAGGCCAACCGTGAAAAGA 3'	5' TGCCTGTGGTACGACCAGAG 3'
Acox1	5' AGGAGGGGATTTT-CTTGAGG 3'	5' AGTCAAAGGCATCCACCAAA 3'
Bhmt1[2]	5′ ATTCAAGTCTGCACAGTAGCCA 3′	5′ ACACAAACACTGACTTTCAGCC 3′
Chdh	5′ CAGTGTGGGCAAGGATGAGTAC 3′	5′ GCAGCAGCACCCGATGATTC 3′
Chrebp1	5' AAGTCCACCCACCTTGGCCT 3'	5' AGGCTCCAGTGCTTGCTCAG 3'
Dgat1	5' GCTTCAAGTGGGGCTGATCC 3'	5' GGGGACCGCCAGCTTTAAGA 3'
Elovl5	5' GGGTGGCTGTTCTTCCAGATTG 3'	5' GTGGCCCTTCAGGTGGTCTTTC 3'
Fads1	5' GCCTTCAACAACTGGTTCAGTG 3'	5' CGTACTTGGCGCACAGGGATTG 3'
Fasn	5′ GGAGTGAAAGTGTCAGATCTGC 3′	5′ CAGTCCCACAGAAGTCAGTAGG 3′
Fatp1	5′ GGCCACCATTCCTACAGCAT 3′	5′ CCACCGTCAACCCGTAGATG 3′
Mttp	5′ GCCACCACTGTTCTCCAGAGAT 3′	5′ TTGTGCGCACCGTCTTCTCATG 3′
Pcyt1a	5′ AGCGAGGATGAAGAGGACTAAC 3′	5′ TGTCCTTAGGTTTAGTGTTGGG 3′
Pemt	5′ TTGGGGATTCGTGTTTGTGC 3′	5′ ACGCTGAAGGGAAATGTGGTC 3′
Scd1	5′ CGCCCCTACGACAAGAACATTC 3'	5′ TGGCAGAGTAGTCGAAGGGG 3′
Srebp1c	5′ CATGGACGAGCTGGCCTTC 3′	5′ ACTGTCTTGGTTGTTGATGAGC 3′

[1] *Actb*: beta-actin; *Acc*: acetyl-CoA carboxylase; *Acox1*: peroxisomal acyl-coenzyme A oxidase 1; *Bhmt*: betaine—homocysteine *S*-methyltransferase; *Chdh*: choline dehydrogenase; *Chrebp1*: Carbohydrate-responsive element-binding protein; *Dgat1*: diacylglycerol O-acyltransferase 1; *Elovl5*: fatty acid elongase 5; *Fasn*: fatty acid synthase; *Fatp1*: fatty acid transport protein 1; *Mttp*: microsomal triglyceride transfer protein; *Pcyt1a*: choline-phosphate cytidylyltransferase A; *Pemt:* phosphatidylethanolamine N-methyltransferase; *Ppara*: Peroxisome proliferator-activated receptor alpha; *Scd1*: stearoyl-CoA desaturase-1; *Srebp1c*: Sterol regulatory element-binding protein 1c

[2] *Bhmt1, Chdh, Pcyt1a,* and *Pemt* primers were previously published [37].
